# Supplementary material for: Reconciling Mining with the Conservation of Cave Biodiversity: A Quantitative Baseline to Help Establish Conservation Priorities
Source: PLoS One. 2016 Dec 20;11(12):e0168348. doi: 10.1371/journal.pone.0168348 (PMC5173368; doi:10.1371/journal.pone.0168348)
Supplement: S1 Dataset — (ZIP) [file pone.0168348.s002.zip › Taxa/Serra Norte/SN_2013/N4WS/Lista N4WS-11.pdf]

CAVIDADE N4WS-0011

Classe	Ordem	Fam/Outros	Gên/Outros	Espécie	S-U	U-S	Zon.
Annelida	Oligochaeta			jovem		4	E/P
Arachnida	Acari	(Oribatida)		sp.3		X	
Arachnida	Acari	Labidostommatidae		sp.	X		
Arachnida	Acari	Trombidioidea		sp.	X		
Arachnida	Amblypygi	Charinidae	*Charinus*	sp.1		2	E
Arachnida	Amblypygi	Phrynidae	*Heterophrynus*	*longicornis*	X	1	E
Arachnida	Araneae	Araneidae	*Pronous*	sp.1	X		
Arachnida	Araneae	Corinnidae		jovem		1	E
Arachnida	Araneae	Ctenidae	*Ctenus*	sp.1		1	E
Arachnida	Araneae	Ochyroceratidae	*Speocera*	sp.1	X	X	P
Arachnida	Araneae	Pholcidae	*Mesabolivar*	sp.1	X	X	E
Arachnida	Araneae	Salticidae		sp.	X		
Arachnida	Araneae	Scytodidae	*Scytodes*	*eleonorae*	1		
Arachnida	Araneae	Theridiidae		jovem		X	E
Arachnida	Araneae	Theridiosomatidae	*Plato*	sp.1	X	X	P
Arachnida	Palpigradi			jovem		X	P
Arachnida	Pseudoscorpiones	Chernetidae	*Spelaeochernes*	sp.1	X	X	E/P
Arachnida	Pseudoscorpiones	Chthoniidae	*Pseudochthonius*	sp.1		X	P
Arachnida	Schizomida	Hubbardiidae	aff. *Rowlandius*	sp.1	X	X	E/P
Chilopoda	Scolopendromorpha	Scolopocryptopidae	*Dinocryptops*	*miersii*	X		
Diplopoda	Polydesmida	Chelodesmidae		jovem		3	E
Diplopoda	Polydesmida	Pyrgodesmidae		sp.1		X	E
Diplopoda	Spirostreptida	Pseudonannolenidae	*Pseudonannolene*	jovem		1	E
Entognatha	Collembola	Isotomidae		sp.1		X	E/P
Entognatha	Collembola	Paronellidae		sp.1		X	E
Entognatha	Diplura	Campodeidae		sp.1	X		
Insecta	Blattodea			jovem	X		
Insecta	Coleoptera	Staphylinidae		sp.61		X	E
Insecta	Diptera	Cecidomyiidae	Cecidomyiinae	sp.	X	X	E
Insecta	Diptera	Culicidae	*Culex*	sp.		X	E
Insecta	Diptera	Psychodidae	Phlebotominae	sp.		X	E
Insecta	Diptera	Tipulidae	Tipulinae	sp.	X	X	E
Insecta	Heteroptera	Reduviidae	*Zelurus*	sp.	X		
Insecta	Homoptera	Cixiidae		jovem		X	E/P
Insecta	Hymenoptera	Formicidae	*Camponotus*	sp.1		X	E/P
Insecta	Hymenoptera	Formicidae	*Pachycondyla*	*harpax*	X		
Insecta	Hymenoptera	Formicidae	*Pheidole*	sp.	X		
Insecta	Hymenoptera	Nyssonidae		sp.2		X	E
Insecta	Lepidoptera	Galacticidae		sp.		X	E
Insecta	Lepidoptera	Tineoidea		sp.	X		
Insecta	Neuroptera	Myrmeleontidae		jovem	X		
Insecta	Orthoptera	Phalangopsidae	*Paraclodes*	sp.	X		
Insecta	Orthoptera	Phalangopsidae	*Phalangopsis*	sp.		3	E/P
Insecta	Psocoptera	Psocomorpha		jovem	X		
Gastropoda	Pulmonata	Subulinidae		sp.2		X	E/P
Malacostraca	Isopoda	Philosciidae		sp.1	X	X	P
Turbellaria				sp.	X		
Amphibia	Anura			sp.		X	P
Mammalia	Chiroptera			sp.	2		E/P
